# Impact of low-level gestational exposure to organophosphate pesticides on neurobehavior in early infancy: a prospective study

**DOI:** 10.1186/1476-069X-12-79

**Published:** 2013-09-13

**Authors:** Kimberly Yolton, Yingying Xu, Heidi Sucharew, Paul Succop, Mekibib Altaye, Ann Popelar, M Angela Montesano, Antonia M Calafat, Jane C Khoury

**Affiliations:** 1 Department of Pediatrics, Division of General and Community Pediatrics, Cincinnati Children’s Hospital Medical Center, Cincinnati, Ohio; 2 Department of Pediatrics, Division of Biostatistics and Epidemiology, Cincinnati Children’s Hospital Medical Center, Cincinnati, Ohio; 3 College of Medicine, Department of Environmental Health, University of Cincinnati, Cincinnati, Ohio; 4 Division of Laboratory Sciences, National Center for Environmental Health, Centers for Disease Control and Prevention, Atlanta, Georgia

**Keywords:** NNNS, Organophosphates, Pesticides, Neurobehavior, Infancy, Prenatal exposure

## Abstract

**Background:**

National data suggest widespread gestational exposure to organophosphate pesticides (OPs) based on the detection of OP metabolites in the urine of pregnant women. Associations with early infant neurobehavior are largely understudied, with only two studies reporting abnormal reflexes in newborns in association with gestational exposure to OPs. Our objective was to utilize biological markers of OP metabolites in pregnant women and a comprehensive assessment of infant neurobehavior to determine the association of gestational exposure to OPs with neurobehavioral outcomes during early infancy.

**Methods:**

Among a cohort of 350 mother/infant pairs, we measured six common dialkylphosphate metabolites of OP pesticides in maternal urine, at two times during pregnancy (16 w & 26 w gestation), then calculated aggregate concentrations of diethylphosphate, dimethylphosphate, and total dialkyphosphate metabolites. We measured infant neurobehavior at about five weeks of age using the NICU Network Neurobehavioral Scale (NNNS), a comprehensive assessment of neurobehavior in young infants. Analyses of associations between gestational exposure to OPs and neurobehavior at five weeks included multiple linear and logistic regression.

**Results:**

After adjustment for covariates, higher creatinine-corrected urinary concentrations of diethylphosphate metabolites were associated with improved attention and reduced lethargy and hypotonia in young infants. Higher creatinine-corrected urinary concentrations of total dialkylphosphate metabolites were associated with fewer signs of autonomic stress. Women who were white, married, had advanced education, and reported more frequent consumption of fresh fruits and vegetables had higher concentrations of OP metabolites during pregnancy.

**Conclusions:**

In this sample of pregnant women whose urinary concentrations of dialkylphosphate metabolites are representative of national exposure levels, we found no detrimental effects of gestational exposure to OPs on neurobehavioral outcomes among young infants. These results are important as they suggest there may be minimal to no detectable adverse impact of low level prenatal OP exposure on the neurobehavior of young infants.

## Background

Organophosphate pesticides (OPs) were banned from U.S. home use in 2000, yet they remain the primary insect control agent in agriculture. The primary route of human exposure to OPs is thus through ingestion of contaminated food. National data suggest widespread gestational exposure to OPs based on the detection of OP metabolites in the urine of pregnant women at rates of 33-83% depending on the specific metabolite tested [1]. Exposure to OPs during pregnancy has been associated with early childhood delays in cognitive abilities [2-4] and impairments in motor skills [4], middle childhood intellectual deficits [5,6], and child behaviors characteristic of attentional disorders [5,7].

Neurobehavioral assessment in early infancy provides a description of an infant’s core neurological make-up, tone and reflexes, coping strategies, and social skills prior to encountering the influences of postnatal growth, life experiences, and additional exposures, and may provide a projection of child functioning many years later. Indeed, neurobehavior in early infancy has been found to be predictive of later outcomes of development, behavior, and intelligence to ages 3 and 4.5 years [8,9]. Early detection of neurobehavioral deficits may allow for implementation of early intervention strategies to improve child outcomes. Assessments such as the Neonatal Behavioral Assessment Scale (NBAS) [10] and the NICU Network Neurobehavioral Scale (NNNS) [11] have been critical in characterizing the early effects of gestational exposure to alcohol [12-17], drugs of abuse [18-23], and tobacco [15,24-27]. These early infant neurobehavioral assessments have been only modestly utilized in environmental exposure research, but they show great promise in helping to illuminate the earliest detectable impact of gestational exposures to environmental toxicants. Such studies have reported associated outcomes related to prenatal exposures to polychlorinated biphenyls [28,29], methyl mercury [30], lead [31,32], phthalates [33,34], organochlorine pesticides [35,36], and OPs [37,38].

Only two studies have specifically examined associations between gestational exposure to OPs and neurobehavior in early infancy. Young et al. [38] and Engel et al. [37] estimated the association between OP exposure during pregnancy, assessed from the urinary concentrations of six dialkylphosphate metabolites, and neurobehavior in early infancy measured with the NBAS. Young et al. [38] evaluated maternal OP exposure at two times during pregnancy (14 w, 26 w) and infant neurobehavior around three days after birth in a Latino agricultural community sample. Women with higher urinary concentrations of dialkylphosphate metabolites during pregnancy had infants with more abnormal reflexes during the NBAS examination. In an urban sample, Engel et al. [37] examined maternal exposure to dialkylphosphates and malathion dicarboxylic acid (MDA), a specific OP with heavy urban use, at around 31 weeks gestational age; infant neurobehavior was assessed around 2 days after birth. Consistent with the findings of Young, women with higher urinary concentrations of MDA and dialkylphosphate metabolites had infants with more abnormal reflexes during the NBAS examination. Pregnant women in both of these samples experienced relatively high exposure to OPs.

Given the widespread exposure to OPs yet limited availability of conclusive studies evaluating their effect on infant neurobehavior, the purpose of the current study was to determine the association of prenatal exposure to OPs with neurobehavior during early infancy, measured with the NNNS, in a sample of women with generally low exposure levels that may be more representative of national exposures levels than the two previous studies of women with high exposures.

## Methods

### Study population

The study population is comprised of women and infants who were enrolled in the Health Outcomes and Measures of the Environment (HOME) Study. This is an ongoing prospective pregnancy and birth cohort study designed to examine the impact of low-level prenatal and early childhood exposure to a variety of environmental toxicants on child health and development. Detailed study eligibility criteria and enrollment methods have been described elsewhere [27,39]. Between March 2003 and February 2006, we enrolled 468 healthy adult (≥18 years) pregnant women in the Cincinnati, Ohio area, and 398 remained in the study and delivered live infants. The cohort is socioeconomically diverse, including urban, suburban, and rural participants. Primary ethics review and approval was provided by the Cincinnati Children’s Hospital Institutional Review Board. In addition, ethics review and approval was provided by several secondary institutions including three hospitals, the Centers for Disease Control and Prevention, and an independent ethics review panel.

For the current study, we excluded the nine sets of twins. Of the remaining 389 singletons, we further restricted the sample to 382 women whose 16-week and 26-week urine samples were collected within four weeks of the target collection point (i.e., 16 w ± 4 w and 26 w ± 4 w) to avoid overlapping time periods that could obscure estimates of time-specific exposure. We then restricted the sample further to 350 women whose infants had neurobehavior examinations performed in the home at approximately 5 weeks of age (mean 34 days, range: 17–47 days).

### Insecticide exposure

Spot urine samples were collected from the mothers at 16 ± 4 and 26 ± 4 weeks gestation and stored in polypropylene containers at −20°C until shipment to the Centers for Disease Control and Prevention (CDC) for analysis. We measured six dialkylphosphates (DAPs) that are metabolites of OPs: dimethylphosphate (DMP), dimethylthiophosphate (DMTP), dimethyldithiophosphate (DMDTP), diethylphosphate (DEP), diethylthiophosphate (DETP) and diethyldithiophosphate (DEDTP), using a modified version of the method described by Bravo et al. [40] Quality control was assessed with standards, blanks, and spiked urine-based quality control materials analyzed with the study samples. The limits of detection (LODs) were 0.6, 0.2, 0.5, 0.6, 0.4 and 0.4 μg/L for DMP, DMTP, DMDTP, DEP, DETP and DEDTP, respectively. The metabolite concentrations were converted from mass concentrations (μg/L) to molar concentrations (nmol/L) and then summed to obtain aggregated concentrations of diethyl phosphates (DE = DEP + DETP + DEDTP), dimethyl phosphates (DM = DMP + DMTP + DMDTP), and DAP total (DAP = DEP + DETP + DEDTP + DMP + DMTP + DMDTP). Creatinine was also measured and used as a correction factor for the dilution of the urine samples.

### Infant neurobehavior

Early infant neurobehavior was measured using the NICU Network Neurobehavioral Scale (NNNS) [11] during a home visit at approximately 5 weeks after birth. The NNNS involves evaluation of neurologic and behavioral qualities of the infant as well as observation of both overt and subtle signs of stress during the exam. The measure is appropriate for infants 30 to 46 weeks gestational age and is especially sensitive to the capabilities and vulnerabilities of high-risk infants such as those born prematurely or prenatally exposed to potentially neurotoxic substances. The NNNS exams were completed by one of four examiners who had been trained to reliability and was blind to infant exposure. Exams lasted about 30 minutes, and the majority (89%) were done with the examiner and infant alone in a quiet room while the mother was engaged in a study interview in a separate area.

The NNNS begins with a baseline observation of respiration, color, and tone. If the infant is asleep, a sequence of habituation items is presented to measure the infant’s ability to process visual, auditory, and tactile stimuli, and to protect sleep. The habituation package is often omitted due to the sleep requirement. Examination of primitive reflexes, as well as passive and active tone ensues, followed by social interaction components and an assessment of attention. Additional neurological items are completed, followed by a post-exam observation of respiration, color, and tone to end the assessment. Summarization of NNNS raw data results in scores on 13 dimensions: habituation, attention, arousal, self-regulation, special handling needed from the examiner to assist the infant through the exam, quality of movement, excitability, lethargy, non-optimal reflexes, asymmetrical reflexes, hypertonicity, hypotonicity, and stress/abstinence. The number of infants with scores on the habituation scale of the NNNS was too small for meaningful interpretation, so we excluded this scale from analyses as we have done in previous work with the NNNS [27,34] and consistent with other researchers [24,26]. The hypotonia, hypertonia, and physiological stress scales were dichotomized due to distributions in which the majority of infants acquired a score of zero and few had values greater than one. Other scales were analyzed as continuous variables. Using latent profile analysis, we identified groupings based on patterns of behavior across the dimensions of the NNNS, allowing for identification of a descriptive categorization of the infant [9]. These profiles were also used in the analyses.

### Measured covariates

For all analyses, we included race and infant age in days at exam as standard covariates. We then examined the following additional covariates for their potential contributions to both OP exposure and neurobehavioral outcome: sex, birthweight, infant weight change from birth to exam, parity, maternal age at delivery, marital status, education, employment during pregnancy, household income, body mass index (BMI) at 16 weeks gestation, weight gain per week during pregnancy, moderate to severe depression (score > 13) measured on the Beck Depression Inventory-II [41] during pregnancy and at 5 weeks postdelivery, reported marijuana and alcohol use during pregnancy, whole blood lead and folate during pregnancy, serum cotinine, and reported fruit and vegetable consumption during pregnancy. Details of measurement and categorization of these covariates are included in Table 1.

**Table 1:** Characteristics of Sample (N = 350)

**Maternal characteristics**	**Frequency (percent)**
Maternal age at delivery (years) ^a^	30 (5.7)
Race	
White, non-Hispanic	223 (64%)
Black, non-Hispanic	104 (30%)
Other	23 (6%)
Marital status	
Married	235 (67%)
Not married, living with someone	48 (14%)
Not married, living alone	67 (19%)
Household income ($) ^b^	55 K (27 K – 85 K)
Employed	288 (82%)
Education	
<= HS or GED	74 (21%)
Some college or college graduate	91 (26%)
College graduate	107 (31%)
Graduate or professional school	78 (22%)
Moderate to severe depression	
(BDI-II > 13 at Baseline or 5-wk post-partum)	89 (25%)
Maternal BMI ^a^	27.5 (6.8)
Weight gain during pregnancy ^a^ (lbs/week)	1.2 (0.5)
Alcohol use during pregnancy	
Never drank alcohol during pregnancy	193 (55%)
Drank <1 alcoholic drink per month	106 (30%)
Drank >1 alcoholic drink per month	51 (15%)
Marijuana use during pregnancy	23 (6%)
Max blood lead (microg/dL) ^c^	0.82 (0.79 – 0.86)
Max serum cotinine (ng/mL) ^c^	0.095 (0.072 – 0.12)
Reported Active smoking during pregnancy	39 (11%)
Folate (total whole blood folate, nmol/L) ^c^	410.8 (117.9–1431.7)
Fresh fruit and vegetable consumption during pregnancy	
≥ daily	215 (61%)
< daily	135 (39%)
Any organic fruit and vegetable consumption	162 (46%)
**Infant characteristics**	
Male	163 (46%)
Gestational age (weeks) ^a^	39 (1.7)
Birthweight (grams) ^a^	3391 (615)
Age at 5-week NNNS exam (days)	34 (5)
Birth order	
First child	153 (44%)
Second child	114 (32%)
> Second child	83 (24%)

^a^ Mean (SD), ^b^ Median (25th, 75th percentile) or ^c^ Geometric Mean (95% confidence interval).

### Statistical analysis

SAS® version 9.3 [42] was used for the analyses. Initially, the data were examined for missing values, distributional properties, and outliers. For any given DAP metabolite concentration below the LOD, the following procedure was used: for concentrations reported as a positive value, that value was used in analyses; for concentrations reported as a zero value or no value, the concentration was imputed. The imputed values were calculated in the following way: 1) for all of the samples, the minimum detectable concentration measured for each metabolite at each visit was determined, 2) the RANUNI function in SAS was used to generate random numbers between zero and one for each of these “missing” values, 3) the imputed concentration was calculated by multiplying the randomly generated number by the minimum valid value determined above for that metabolite. We applied creatinine correction by dividing the molar DE, DM, and DAP metabolite concentrations by the creatinine concentration. The independent variables for the analyses were the log base 2 transformed values of the creatinine corrected DE, DM, and DAP at 16 and 26 weeks, as well as the mean of log transformed values of 16 and 26 week concentrations. For the 350 participants included in the analysis, 320 had dialkylphosphate measures at both the 16- and 26-week time points, 6 had missing measures at 16 weeks, and 24 had missing measures at 26 weeks.

We tested associations between the independent variables (16-week, 26-week, and mean DE, DM, DAP), the dependent variables (NNNS scales), and the potential covariates using Pearson correlation, analysis of variance (ANOVA), and Chi-square, as appropriate. When the level of significance for the association of dialkylphosphates and NNNS scales was <0.10 in the bivariate analyses, we included those covariates in a subsequent multiple linear or logistic regression analyses, as appropriate. Specifically, as hypotonia, hypertonia, and physiological stress scales were dichotomized as described above, multiple logistic regression was used for analysis, while multiple linear regression was used for the analysis of the rest of the outcome variables. Backward elimination of variables was used to determine the most parsimonious models. A priori, we decided to retain race and infant age in days at exam in all models, based on our previous work. [27,34] Other covariates were retained in the final model if they were statistically significant (p < 0.05) or if the beta coefficient for the independent variable of interest was changed by more than 10% by removal of the covariate from the model.

We conducted a secondary analysis using groupings obtained from latent profile analysis (LPA) [9] including the same potential covariates as described above. Briefly, infants with similar patterns of NNNS scale scores were grouped together within one of three latent profiles labeled as social/easy going, hypotonic, and high arousal/difficult based on the description of the NNNS response patterns of each profile. Using logistic regression, we evaluated the association between the OP exposure variables of interest and risk of being classified in the high arousal/difficult profile and hypotonic profile versus the social/easy going profile, separately.

## Results

A total of 350 singleton infants had NNNS examinations conducted at about 5 weeks (mean 34 days). Characteristics of the study sample are shown in Table 1. The mean maternal age at delivery was 30 years, 30% of the mothers were African American, and 67% were married. The mean household income was $55,000, and 82% were employed with 79% having at least some college education. Moderate to severe depression was detected in 25% of the mothers either during pregnancy or at five weeks post-partum. Daily or more frequent fresh fruit and vegetable consumption was reported by 61% of the women. The infants were born at a mean of 39 weeks of gestation with a mean birthweight of 3391 grams.

Table 2 summarizes the creatinine corrected dialkylphosphate urinary concentrations during pregnancy (16 w, 26 w) and the mean of the two sampling points given by the summary DE, DM, and DAP measures. Pearson correlations between the log_2_ of the creatinine corrected summary DE and DM measures acquired at the 16 week, 26 week, and mean of the two time points were 0.43, 0.39, and 0.42, respectively. Pearson correlations between the log_2_ of the creatinine corrected summary DE and DM measures acquired at the 16 week, 26 week, and mean of the two time points were 0.43, 0.39, and 0.42, respectively. Based on the mean summary measures, the percent of detectable dialkylphosphate metabolite concentrations for our sample (93% and 100% for DE and DM, respectively) are comparable to percentages reported by both Young [38] (99.8% for both DE and DM) and Engel [37] (88.8% and 90.2% for DE and DM, respectively). However, based on concentrations without creatinine correction, the median values of mean dialkylphosphate measures for gestational weeks 16 and 26 for the current study (9, 40, 63 nmol/L for DE, DM, and DAP, respectively) are lower than those reported by both Young (22, 101, 136 nmol/L, respectively) and Engel (24.7, 90.2, 96.5 nmol/L, respectively). The geometric mean of DMTP for our study was comparable to the U.S. National Health and Nutrition Examination Survey 2003–2004 general population data [43] at 16 weeks (2.09 vs. 2.06 μg/L); while slightly lower at 26 weeks (1.60 vs. 2.06 μg/L). There are no national reference data available on the summary measures of DE, DM, and DAP for comparison with our cohort.

**Table 2:** Creatinine corrected dialkylphosphate metabolite urinary concentrations in home study maternal samples

		**% > LOD **^**a**^	**Geomean**	**95% CI of Geomean**
**Measures with creatinine correction (nmol/g creatinine)**				
**16-week**	DE	82	10.9	8.9 – 13.3
	DM	94	49.6	40.9 – 60
	DAP	99	78.8	67.1 – 92.7
**26-week**	DE	69	7.9	6.2 – 10
	DM	93	44.6	37.4 – 53.2
	DAP	97	69.2	58.8 – 81.4
**mean**	DE	93	9.4	8 – 11.1
	DM	100	46.4	40.2 – 53.7
	DAP	100	73.5	64.8 – 83.4

^a^Aggregate measure was considered > LOD if at least one of the component metabolites was detectable (i.e. if any of 16-week DEP, DETP, and DEDTP was > LOD then 16-week DE was considered LOD).

Table 3 summarizes the associations between various descriptive characteristics of our cohort and creatinine corrected urinary concentrations for DE, DM, and DAP metabolites as determined by ANOVA. These results indicate that women who were white, married, with more advanced education, and who reported at least daily consumption of fresh fruits and vegetables had higher concentrations of DE, DM, and DAP than other women. In addition, dose–response trends were observed with respect to marital status and education. Women who were single and living alone had the lowest dialkylphosphate metabolite concentrations, followed by those who were single with a live-in partner, and those who were married had the highest concentrations. Similarly, women with a high school degree or some college had the lowest concentrations, followed by college graduates, and those with graduate or professional degrees had the highest concentrations. Women who reported consumption of any organic produce had higher concentrations of DM and DAP than women who reported no organic produce use.

**Table 3:** Creatinine corrected dialkylphosphate metabolite urinary concentrations (mean of 16w and 26w measures) by maternal and child characteristics

**Characteristic**	**Value**	**N**	**DE**		**DM**		**DAP**	
			**G-Mean **^**a**^	**95% CI**	**G-Mean **^**a**^	**95% CI**	**G-Mean **^**a**^	**95% CI**
**Sex**	Female (ref)	187	9.0	7.1 – 11.4	46.3	37.8 – 56.6	71.1	59.5 – 84.9
	Male	163	9.9	7.9 – 12.4	46.6	37.8 – 57.4	76.4	64 – 91.3
**Race**	Non-Black (ref)	246	10.7	8.8 – 13.0	55.6	47.1 – 65.5	87.7	76.2 – 101
	Black	104	6.9 *	5.1 – 9.4	30.3 *	23 – 40	48.4 *	37.9 – 61.8
**Maternal education**	≤ HS graduate (ref)	74	6.6	4.6 – 9.4	27.9	20 – 38.9	44.7	33.7 – 59.4
	Some college	91	6.3	4.4 – 9	33.3	26.1 – 42.4	51.8	41.5 – 64.7
	College graduate	107	11.4 *	8.5 – 15.3	65.9 *	52 – 83.5	101.3 *	82 – 125
	Graduate/Professional degree	78	16.1 *	12.4 – 21.1	68.5 *	49.4 – 95.1	114.1 *	88.5 – 147.2
**Marital status**	Married (ref)	235	11.4	9.4 – 13.8	58.1	49.2 – 68.8	91.3	79 – 105.6
	Not married, living with partner	48	7.0	4.3 –11.6	25.0 *	16.9 – 37.1	44.5 *	31.1 – 63.6
	Not married, living alone	67	5.9 *	4.0 –8.5	32.8 *	23.3 – 46.2	49.2 *	37.0 – 65.5
**Maternal employment**	Not employed (ref)	62	6.8	4.3 – 10.5	32.9	23.4 – 46	51.8	37.8 – 71.1
	Employed	288	10.1	8.5 – 12	50.0 *	42.6 – 58.6	79.3 *	69.2 – 90.8
**Fresh fruit & vegetable intake**	< Daily (ref)	135	7.0	5.3 – 9.2	33.6	26.8 – 42.1	53.7	44 – 65.4
	≥ Daily	215	11.3 *	9.3 – 13.9	56.9 *	47.4 – 68.3	89.6 *	76.5 – 104.9
**Organic Use**	None (ref)	188	8.6	6.8 – 10.8	38.0	31.1 – 46.5	63.1	53 – 75.1
	Any	162	10.4	8.2 – 13.2	58.5 *	47.7 – 71.7	87.8 *	73.4 – 105

^a^ G-Mean = Geometric Mean in nmol/g creatinine, * indicates significant difference compared to reference group (p < 0.05).

In bivariate analyses of dialkylphosphate metabolite concentrations and NNNS scales, higher maternal creatinine corrected DE urinary concentrations were associated with increased infant attention and physiological stress, and decreased lethargy, hypotonia, asymmetry, and autonomic stress at p < 0.10. Higher maternal DM urinary concentrations were associated with infant decreased infant CNS stress and increased infant visual stress at p < 0.10. Higher maternal DAP urinary concentrations were associated with decreased infant autonomic stress and CNS stress at p < 0.10. These subscales were further evaluated with multivariable linear or logistic regression including covariates in the model. Table 4 shows the multiple regression results for NNNS subscales whose beta coefficients for creatinine corrected maternal dialkylphosphate metabolites were statistically significant at p < 0.05.

**Table 4:** Models with significant creatinine corrected dialkylphosphate metabolite coefficients at p ≤ 0.05

**OP**	**NNNS subscale**	**Exposure window**	**Adjusted R**^**2**^	**Covariate**	**Coeff**	**SE**	**p-value**
**DE**	**Attention**	Mean	0.011		0.066	0.033	<0.05
				Black race	−0.109	0.166	0.51
				Age (days)	−0.020	0.015	0.18
	**Lethargy**	16 week	0.030		−0.069	0.034	0.04
				Black race	−0.462	0.212	0.03
				Age (days)	0.006	0.019	0.76
				Birthweight (g)	−0.044	0.016	0.01
				Fresh fruit/veg	−0.346	0.195	0.08
	**Hypotonia (0/1)**	16 week	0.037		−0.101	0.045	0.03
				Black race	−0.243	0.295	0.41
				Age (days)	−0.050	0.026	0.05
				Body mass index	0.038	0.019	0.04
**DAP**	**Autonomic stress**	26 week	0.039		−0.010	0.004	0.01
				Black race	−0.014	0.020	0.47
				Age (days)	−0.002	0.002	0.14
				Birthweight (g)	−0.003	0.001	0.02
				Blood lead	0.031	0.014	0.02

With adjustment for covariates, we found several statistically significant associations among DE and DAP measured during pregnancy and infant performance scores from the NNNS subscales. For DE, 1) an increase in the mean creatinine corrected DE was associated with an increase in attention, no covariates contributed significantly to the association; 2) an increase in creatinine corrected DE at 16 weeks was associated with decreased lethargy, with Black race and birthweight as statistically significant covariates in the model; 3) an increase in creatinine corrected DE at 16 weeks was associated with decreased hypotonia, with infant age (days) and maternal body mass index at about 16 weeks gestation contributing significantly to this association. For DAP, an increase in 26-week creatinine corrected DAP was associated with decreased autonomic stress. Infant birthweight and maternal blood lead level during pregnancy were statistically significant covariates for this association. No significant associations were found between DM and performance scores on the NNNS scales.

### Analysis of latent profile groups

The descriptions of the individual NNNS subscales contained within the three profiles are detailed elsewhere [9]. Briefly, three profiles emerged describing infants who were generally social/easy going (45%, n = 157), high arousal/difficult (31%, n = 83), or hypotonic (24%, n = 110) during the NNNS assessment. Results of the logistic regression models comparing infants in the high arousal/difficult profile and the hypotonic profile to the social/easy going profile by the creatinine corrected dialkylphosphate urinary metabolite concentration during pregnancy are shown in Table 5. In addition to the standard covariates of race and age in days at exam, maternal weight gain per week during pregnancy and BMI at 16 weeks remained in the models due to significant associations. The models comparing the high arousal/difficult profile to the social/easy going profile showed no significant associations with dialkylphosphate urinary metabolite concentrations before or after adjustment for covariates. For the models comparing the hypotonic profile to the social/easy going profile, infants were less likely to be classified as hypotonic if mothers had higher concentrations of DE at 16 weeks (OR = 0.89 95% CI: 0.81, 0.99, p = 0.03) with adjustment for covariates.

**Table 5:** Prediction of NNNS profile membership with creatinine corrected dialkylphosphate metabolite urinary concentrations

**OP**	**NNNS profile **^**a**^	**Mean OR (95% CI)**	**16 Week OR (95% CI)**	**26 Week OR (95% CI)**
**DE**	High Arousal/Difficult	1.03 (0.92, 1.15)	0.98 (0.89, 1.08)	1.03 (0.95, 1.12)
	Hypotonic	0.96 (0.86, 1.09)	0.89 (0.81, 0.99) *	1.03 (0.94, 1.13)
**DM**	High Arousal/Difficult	1.11 (0.97, 1.26)	1.00 (0.90, 1.10)	1.12 (1.00, 1.25)
	Hypotonic	0.99 (0.86, 1.13)	0.90 (0.80, 1.00)	1.12 (0.99, 1.26)
**DAP**	High Arousal/Difficult	1.14 (0.98, 1.32)	1.02 (0.91, 1.15)	1.13 (0.99, 1.27)
	Hypotonic	1.02 (0.87, 1.19)	0.90 (0.79, 1.03)	1.13 (0.99, 1.29)

^a^Social/easy going profile is the reference group, * p < 0.05.

Covariates: Black race and age at exam were included in all models, regardless of significance. Maternal weight gain per week during pregnancy and BMI also remained in the models due to significant associations. All other potential covariates were not significant.

## Discussion

In this study, we report associations between gestational exposure to OPs and neurobehavior in early infancy that suggest increased attention, and reductions in lethargy, hypotonic responses, and signs of autonomic stress with higher exposure. More specifically, higher urinary concentrations of DE metabolites as measured by the mean of the 16- and 26-week maternal samples were associated with improved attention in infants. Higher urinary concentrations of DE metabolites measured at 16 weeks gestation were also associated with reduced lethargy in the infants as well as a reduction in hypotonia and hence improved muscle tone. Higher urinary concentrations of DAP metabolites measured at 26 weeks gestation were associated with a reduction in infant signs of autonomic stress such as spitting up, sneezing, and hiccoughing during the assessment. All significant findings from multiple regression analyses indicate improved performance from the infant with greater exposure to OPs during gestation. Consistent with these findings, when using the groups from our original LPA on this cohort, we also found that infants were less likely to be classified as hypotonic, compared with social/easy going, if their mothers had higher concentrations of DE at 16 weeks. There were no other statistically significant associations with OP exposure by LPA group.

We are extremely cautious in interpreting these findings as evidence of actual benefits of exposure to OPs during gestation. The neurotoxic nature of OPs by design, and as reported in previous studies of acute and chronic exposure, lead us to two possible explanations. First, the levels of exposure to OPs in our sample may be below a threshold at which detrimental effects are realized. The urinary concentrations of dialkylphosphate metabolites in our cohort are indeed substantially lower than those reported in the two previous studies that reported a detrimental impact of OP exposure on infant neurobehavioral outcome [37,38]. Lu found that both OP parent compounds and dialkylphosphate metabolites could be measured in some fresh fruit juices [8]. This suggests that OPs may independently degrade resulting in consumption of both the parent compound and metabolites and potentially inflating the metabolite levels measured in urine [44]. It is thus possible that we have measured direct exposure to the dialkylphosphate metabolites among mothers in our cohort in addition to OP parent compounds [43]. If this is indeed the case, the actual exposure to toxic OP parent compounds may have been even lower than the levels we measured. While no studies have yet reported a threshold at which OPs are not harmful to humans, in the U.S., maximum allowable limits of pesticide content in produce are set by the Environmental Protection Agency [45]. It is possible that our cohort was exposed at levels below these recommended limits, and we thus may actually be seeing evidence of a threshold at which these specific OPs have no negative impact on infant neurobehavior. The urinary concentrations in our cohort closely mirror the levels reported nationally [43]; our findings may thus offer some comfort to those who are concerned about pesticide exposure through fresh produce consumption.

Second, it is likely that any detrimental effects of OPs are confounded by socioeconomic status (SES) and nutritional factors in this cohort. Indeed, when we more closely examined the characteristics of the mothers with the highest urinary dialkylphosphate concentrations during pregnancy, we found they were more likely to be white, married, and educated. They also reported more fresh fruit and vegetable consumption than those with lower concentrations. These associations have led us to consider the possibility that greater SES and nutritional advantage may provide added protection to the developing fetus against potential neurobehavioral damage that may arise from gestational OP exposure. These benefits may then outweigh the potential harm at the lower ranges of exposure to the toxicant. This could be likened to the issue of negative confounding that has been associated with findings relating seafood and fish intake with methylmercury exposure [46]. In this association, the essential nutrients in fish that appear to benefit the cardiovascular system and brain development are in competition with the detrimental effects that arise from higher exposure to the methylmercury toxicant that bioaccumulates in fish. The result of this confounding relationship is that at low levels of exposure, there is an appearance that prenatal exposure to methylmercury may have benefits to cognitive function when adverse effects of methylmercury are masked by the beneficial nutrients of fish. At some unspecified higher level of exposure, the influence of methylmercury as a neurotoxicant would reach a tipping point, and harm would be detectable.

Similarly, several studies have demonstrated the protective effects of maternal diet during pregnancy on subsequent child outcomes. Reported maternal fruit and vegetable consumption during pregnancy has been linked with increased fetal growth [47] as well as protective effects against the development of specific brain tumors [48] and the incidence of retinoblastoma [49] in childhood. More specifically related to protection against environmental exposures are studies indicating that consumption of fresh fruits and vegetables containing vitamin C during pregnancy appear to protect against oxidative stress associated with exposure to polycyclic aromatic hydrocarbons [50] and reduce the impact of air pollution on cognitive development in infants [51]. Nevertheless, when we attempted to adjust for dietary and SES factors in our multivariable models, there was no significant impact in nearly all instances. SES factors were not retained in any final models, and only in the model for the lethargy subscale did fruit and vegetable intake demonstrate a trending influence toward lower lethargy. We contend that the benefits of the SES and dietary characteristics among our sample have protected the infants from the potential harm of OP exposure. At some level, we would expect the harm of exposure to outweigh the benefits of these other factors.

Our findings are not in accord with those of Young et al. [38] or Engel et al. [37] who reported an increase in abnormal reflexes with higher gestational exposure to OPs. We suspect that the differences may be related in part to the levels and types of exposure among these very different cohorts that consist of Mexican-American agricultural workers in California [38] and NYC inhabitants [37]. Both of these studies were conducted prior to the ban on household use of OPs, and mothers in both of these cohorts experienced heavier exposure than our mid-west cohort. Because our study occurred after the ban, we expect that the OP metabolite levels are largely due to dietary, rather than agricultural, exposure.

### Limitations

Limitations to this study mainly revolve around collection of the biological samples to characterize maternal exposure to OPs during pregnancy, and thus fetal exposure during gestation. We obtained spot urine samples at two points during pregnancy. Although this is improved over other studies that have collected only one sample during pregnancy, the risk of exposure misclassification remains, given the short half-life of dialkylphosphate metabolites. In future studies, more frequent collection of urine samples spanning all three trimesters of pregnancy may minimize exposure misclassification and better characterize gestational exposure. An additional limitation of this study relates to the narrow range and relatively low concentrations of dialkylphosphates in our cohort. While these factors may somewhat hinder our ability to detect neurobehavioral differences related to prenatal exposure, the urinary concentrations of these metabolites are reflective of those reported nationally and may thus increase our generalizability and relevance to a large percentage of the population.

## Conclusions

In this sample of young infants born to women who had urinary concentrations of dialkylphosphate metabolites of OP insecticides that are representative of the background exposure among U.S. adult females, we found no evidence of detrimental effects of gestational exposure to OPs on neurobehavioral outcomes. These results are important as they suggest there may be no detectable impact of low level prenatal exposure on the neurobehavior of young infants, and that at low levels, the associated benefits of improved SES and dietary qualities may actually provide the illusion that OPs are themselves beneficial. It is important to note that infants who experienced greater exposure to OPs or their degradates were born to mothers who were white and married, had achieved higher education, had higher income, and consumed more fresh fruits and vegetables during pregnancy. Thus, the associations between OP exposure and infant neurobehavior may be difficult to untangle from the added benefits of higher SES and improved nutrition.

## Abbreviations

ANOVA: Analysis of variance; BMI: Body mass index; BNBAS: Brazelton neonatal behavioral assessment scale; CDC: Centers for Disease Control and Prevention; DAP: Sum of all dialkylphosphate metabolites; DE: Sum of diethylphosphates; DEDTP: Diethyldithiophosphate; DEP: Diethylphosphate; DETP: Diethylthiophosphate; DM: Sum of dimethylphosphates; DMDTP: Dimethyldithiophosphate; DMP: Dimethylphosphate; DMTP: Dimethylthiophosphate; HOME Study: Health outcomes and measures of the environment study; LPA: Latent profile analysis; MDA: Malathion dicarboxylic acid; NNNS: NICU Network neurobehavioral scale; LOD: Limit of detection.

## Competing interests

All authors declare that they have no competing financial or non-financial interests with regard to the research described. The findings and conclusions in this report are those of the authors and do not necessarily represent the views of the Centers for Disease Control and Prevention.

## Authors’ contributions

Study conceptualization and design: KY; Data collection: KY, AP; Data cleaning and discrepancy checks: YX, AP, KY, JK; Analysis of biological samples: AMM, AMC; Analytic strategy: YX, HS, PS, MA, JK; Analysis of data: YX, HS, JK; Interpretation of data: KY, YX, HS, JK; Manuscript preparation: KY, YX, HS, PS, MA, AP, JK; Final approval of manuscript: KY, YX, HS, PS, MA, AP, AMM, AMC, JK. All authors read and approved the final manuscript.
